# Synchronous occurrence of gastrointestinal stromal tumor and intrahepatic cholangiocarcinoma: A case report

**DOI:** 10.3892/ol.2014.2703

**Published:** 2014-11-12

**Authors:** SEUNG-JOO NAM, HYUK SOON CHOI, EUN SUN KIM, BORA KEUM, YOON TAE JEEN, HOON JAI CHUN

**Affiliations:** Division of Gastroenterology and Hepatology, Department of Internal Medicine, Korea University College of Medicine, Seoul 136-705, Republic of Korea

**Keywords:** gastrointestinal stromal tumor, cholangiocarcinoma, multiple primary tumors

## Abstract

Various cases of gastrointestinal stromal tumor (GIST) coinciding with other gastrointestinal malignancies have been reported to date, however, the synchronous occurrence of GIST and intrahepatic cholangiocarcinoma (ICC) is exceptionally rare and, to the best of our knowledge, has only been reported once. The coinciding malignancy has usually been encountered incidentally during surgical exploration. Thus, this is the first report where a targeted biopsy of the clinically suspicious lesion was used to determine the diagnosis of ICC concurrent with GIST. The liver is the most frequent metastatic site of GIST, therefore, additional hepatic masses may be mistakenly diagnosed as metastatic disease, rather than the presentation of multiple primary tumors. This subsequently delays the accurate diagnosis and complicates the performance of a curable resection. The current study reports a case of advanced synchronous GIST and ICC, which was operable at initial presentation, but progressed to become surgically unresectable.

## Introduction

Various cases of synchronous gastrointestinal stromal tumors (GIST) and other primary gastrointestinal cancers have previously been reported (1–10). The majority of the published cases reported the synchronous occurrence of GIST with gastrointestinal epithelial malignancies, such as gastric or colorectal adenocarcinoma (1–4, 6–9). The occurrence of synchronous GIST and an intrahepatic malignancy is particularly rare and, to the best of our knowledge, only one case has been reported regarding the synchronous presentation of GIST and intrahepatic cholangiocarcinoma (ICC) (10–12). Furthermore, as the liver is the most common site for GIST metastasis, the symptoms on presentation may be misleading, rendering the early diagnosis of synchronous GISTs and other primary intrahepatic neoplasms difficult (13,14). To the best of our knowledge, all reported cases of synchronous GIST and other primary neoplasms were identified incidentally during surgery; no previous cases have been diagnosed by the targeted biopsy of a clinically suspicious lesion. The current study reports the case of targeted biopsy-identified ICC concurrent with GIST. Written informed consent was obtained from the patient.

## Case report

**Figure 1:**
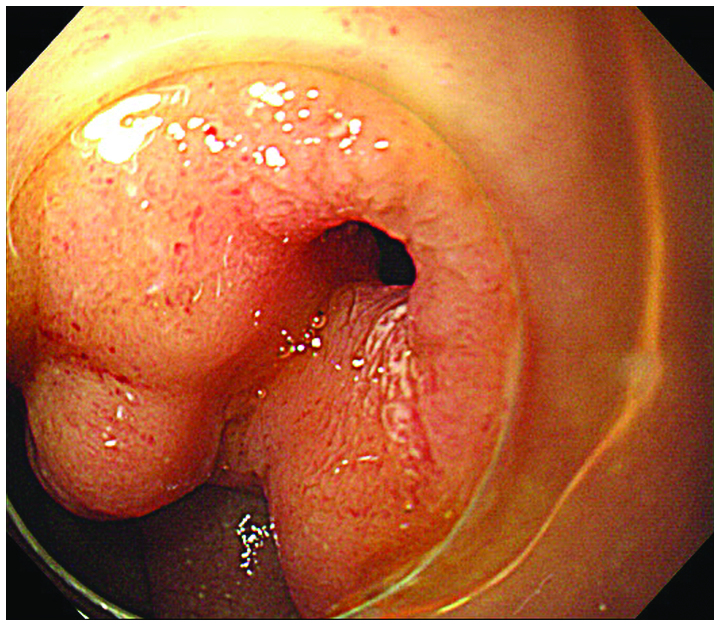
Esophagogastroduodenoscopy upon admission to hospital showing a large, lobulated subepithelial lesion with a fistulous hole in the second portion of the duodenum.

**Figure 2:**
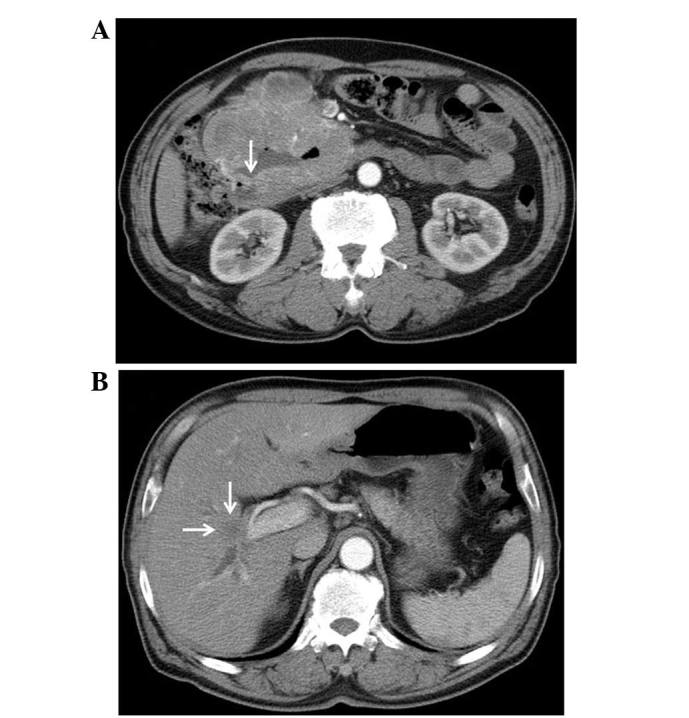
Computed tomography scan of the abdomen upon admission to hospital. (A) Large lobulated and heterogeneously enhanced mass (11.5×9.3 cm) with internal necrosis. An air-fluid level was noted on the pancreaticoduodenal groove and the internal cavity of the mass was connected to the second portion of the duodenum (arrow). (B) An Ill-defined low density lesion was noted at hepatic segment eight, abutting the bile duct and hepatic artery. The right intrahepatic duct was marginally dilated by the lesion.

**Figure 3:**
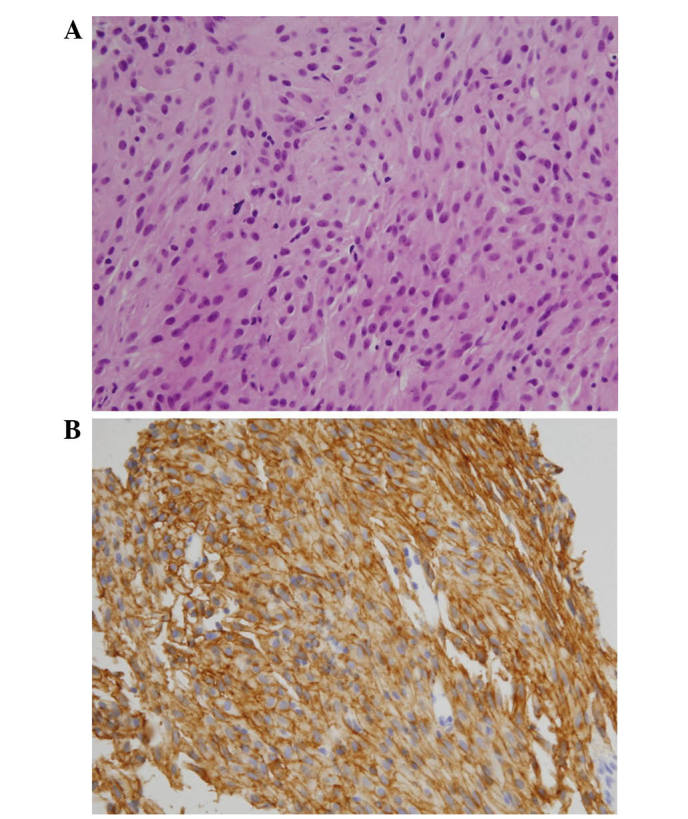
Histopathological findings of the duodenal mass. (A) Hematoxylin and eosin staining shows whirling sheets of spindle-shaped cells, which are consistent with gastrointestinal stromal tumor (magnification, ×40). (B) Immunohistochemical staining demonstrates that the tumor cells were positive for c-Kit (CD117).

**Figure 4:**
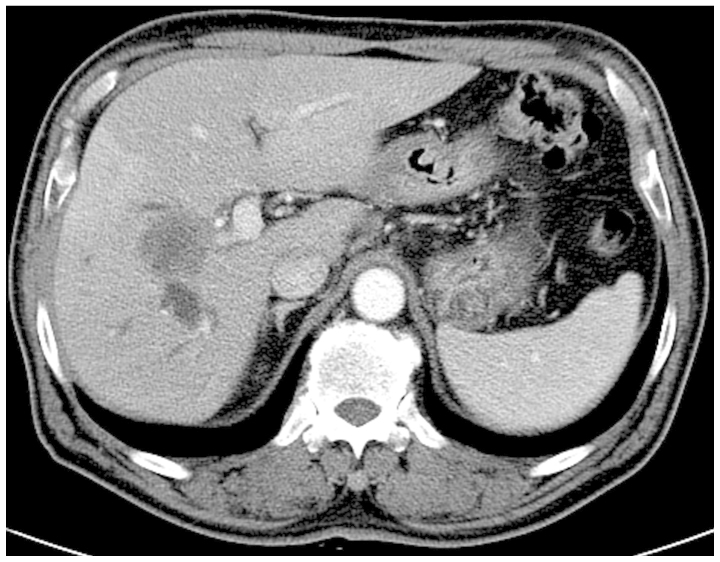
Computed tomography scan of abdomen following seven weeks of imatinib treatment. The previously noted hepatic mass had increased in size and the right intrahepatic duct dilatation was aggravated by the mass.

**Figure 5:**
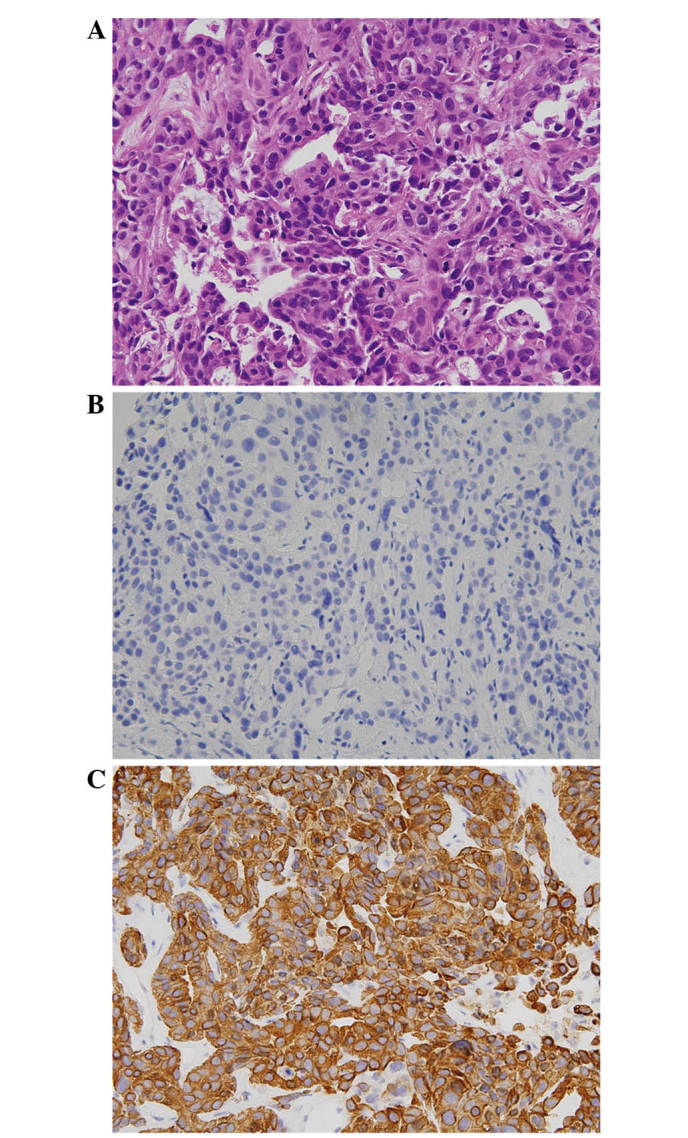
Histopathological findings of the hepatic mass. (A) Hematoxylin and eosin staining shows gland-forming malignant cells with hyperchromatic and prominent nuclei, which is consistent with a moderately differentiated adenocarcinoma (magnification, ×40). Immunohistochemical staining demonstrates that the tumor cells were (B) negative for c-Kit and (C) positive for cytokeratin 19.

In January 2013, a 65-year-old male presented to Korea University Anam Hospital (Seoul, Republic of Korea), with an intra-abdominal mass that was localized to the right side. The mass had presented one month previously. In addition, the patient had experienced weight loss (5 kg) during the previous four months. The medical history was unremarkable, with the exception of pulmonary tuberculosis 30 years previously, from which the patient had recovered. On physical examination, a round mass was palpated and there was tenderness of the right upper quadrant area. Hematological examination revealed a marginal decrease of hematocrit (35.8%; normal range, 37–51%) and hemoglobin (11.8 g/dl; normal range, 12.6–17.4 g/dl). Liver function tests revealed elevated alkaline phosphatase (423 IU/l; normal range, 30–120 IU/l) and γ-glutamyl transferase (587 IU/l; normal range, 9–64 IU/l) levels. Aspartate aminotransferase, alanine aminotransferase and bilirubin levels were within the normal ranges of 3–45 IU/l, 3–45 IU/l and 0.0–0.4 mg/dl, respectively. An upper gastrointestinal endoscopy revealed a subepithelial mass with a fistulous hole on the second portion of the duodenum (Fig. 1). A total colonoscopy revealed no abnormalities. A computed tomography (CT) scan, which was acquired from the referring local clinic (Choi Kang Sik Internal Medicine, Seoul, Republic of Korea), demonstrated a large, heterogeneously enhanced lobulated mass (11.5×9.3 cm; longest diameter × greatest perpendicular diameter) with internal necrosis at the pancreaticoduodenal groove (Fig. 2A). The internal cavity of the mass was connected to the second portion of the duodenum, which was consistent with the endoscopic findings. Furthermore, an ill-defined low-attenuation lesion was identified at segment eight of the liver, abutting the bile duct and hepatic artery (Fig. 2B). Significantly enlarged lymph nodes were not observed in the abdominal cavity. In addition, a fludeoxyglucose positron emission tomography (FDG-PET) scan also revealed hypermetabolic masses in the duodenal groove and in segment eight of the liver, which was consistent with the CT scan. Abdominal MRI (magnetic resonance imaging) was also conducted for further characterization of the duodenal and hepatic masses observed on the CT scan, which confirmed the same findings. An ultrasound-guided needle biopsy of the duodenal mass was performed and pathological examination identified whirling sheets of spindle-shaped cells (Fig. 3A). Immunohistochemical staining was positive for c-Kit, but negative for cluster of differentiation 34, S-100 and desmin (Fig. 3B). Mitotic features could not be evaluated, as a surgically excised sample was not obtained. The diagnosis of high-risk GIST with hepatic metastasis was determined and the patient was treated with 400 mg imatinib, daily. The treatment was tolerated well, and the abdominal mass and distension improved significantly. After seven weeks of treatment, the follow-up abdominal CT scan revealed that the duodenal mass had significantly reduced in size (7.9×6.5 cm; longest diameter × greatest perpendicular diameter) and exhibited an increased area of internal necrosis. However, the hepatic mass had increased from 1.7×1.5 cm to 3.9×3.2 cm in diameter (longest diameter × greatest perpendicular diameter) and the right hepatic duct was markedly dilated by the mass (Fig. 4). Various enlarged lymph nodes were observed in the left gastric area, including the porta hepatis and portocaval space. The ultrasound-guided needle biopsy was repeated for the hepatic mass and histopathological examination of the biopsy specimen showed malignant cells with a glandular structure, which was consistent with adenocarcinoma. Immunohistochemical analysis exhibited c-Kit-negative and cytokeratin 19-positive staining (Fig. 5). The final diagnosis was synchronous ICC and GIST. Two weeks after the ultrasound-guided liver biopsy, the patient developed jaundice and a fever, and the total level of bilirubin increased rapidly to 9.4 mg/dl (normal range, 0.0–0.4 mg/dl). Percutaneous transhepatic biliary drainage was conducted along with antibiotic treatment and administration of imatinib was withheld to allow the patient to recover from the condition. An abdominal CT scan following three weeks of conservative treatment revealed an increase in size of the liver mass (5.3×3.9 cm; longest diameter × greatest perpendicular diameter) with portal vein thrombosis, and the size of the duodenal mass had also increased to 9.5×8.7 cm (longest diameter × greatest perpendicular diameter). The treatment regimen became focused towards the ICC, which was associated with a poorer prognosis. Due to the well-known toxicity of combined chemotherapy, administration of imatinib was terminated and the patient was treated with intravenous gemcitabine (100 mg/m^2^ for 30 min, days 1 and 8) and cisplatin (25 mg/m^2^ for 1 h, days 1 and 8) (15,16) every three weeks for four cycles. During chemotherapy, the abdominal mass reappeared, as a follow-up CT scan, performed six weeks following treatment, revealed a prominent increase in the size of the duodenal mass (10.7×10 cm; longest diameter × greatest perpendicular diameter). However, the hepatic mass did not demonstrate a significant change in size during that interval. As the patient had tolerated the initial chemotherapy well, combined chemotherapy (consisting of imatinib, gemcitabine and cisplatin) was initiated for symptom control and treatment of the GIST. The addition of imatinib resulted in the duodenal mass decreasing significantly and the patient exhibited a good response to the treatment. However, certain toxicities are associated with combined chemotherapy, such as grade 1 bone marrow suppression, as observed in the present case. The follow-up CT scan revealed disease progression of the ICC, whereas the size of the GIST mass had decreased. The treatment modality was altered, due to refractory cholangiocarcinoma, from chemotherapy to radiation therapy (daily dose of 180 Gy), whilst maintaining the imatinib treatment. To date, the patient has been undergoing treatment for one year and is tolerating the treatment well.

## Discussion

Synchronous ICC and GIST is a particularly rare condition and, to the best of our knowledge, only one case has been reported, which was treated via curative surgical resection (10). The current study reports the first case diagnosed by an ultrasound-guided targeted biopsy of a clinically suspicious lesion. The synchronous ICC and GIST was operable on admission, however, subsequently progressed to a surgically unresectable status. Initially, an incorrect diagnosis of GIST metastases was determined; this was due to the liver being the most common site of GIST metastasis, therefore, this was assumed. An accurate diagnosis could only be determined following histopathological examination of the hepatic mass, in addition to the imaging that was initially conducted, including CT, magnetic resonance imaging and FDG-PET. In the case of multiple metastatic malignancies, the possibility of multiple primary tumors must also be considered, particularly when discrepancies in the responses to chemotherapy are observed between the tumors. In the present case, although a liver biopsy was conducted immediately following the discordant response to the imatinib chemotherapy, on determination of the correct diagnosis it was too late for the patient to undergo a curable resection of the ICC. Thus, following imatinib chemotherapy, it may be beneficial to conduct the imaging follow-up earlier than the currently recommended three-month period, which was proposed for metastatic GIST patients by the National Comprehensive Cancer Network consensus panel (17), as demonstrated in the present case. An FDG-PET scan, which indicates a diminished uptake of FDG within a few days of treatment, may be one option for monitoring early therapeutic responses to imatinib (14).

In conclusion, a rare case of synchronous duodenal GIST and ICC is presented, which was diagnosed by a targeted biopsy of the suspected lesion, however, following several weeks of GIST treatment, the patient was subsequently treated with a combined chemotherapy regimen. Although the presentation of the two primary tumors, GIST and an intrahepatic malignancy, is a rare condition and the liver is the most common metastatic site of GIST, the possibility of multiple primary tumors must be considered as an alternative diagnosis.
